# Accessibility of eHealth Before and During the COVID-19 Pandemic Among People With and People Without Impairment: Repeated Cross-Sectional Survey

**DOI:** 10.2196/64707

**Published:** 2025-03-28

**Authors:** Linda Pettersson, Stefan Johansson, Ingrid Demmelmaier, Lena von Koch, Jan Gulliksen, Per-Olof Hedvall, Karl Gummesson, Catharina Gustavsson

**Affiliations:** 1 Center for Clinical Research Dalarna, Uppsala University, Nissers väg 3, Falun, SE-79182, Sweden, 46 23-49 00 00; 2 Department of Public Health and Caring Sciences, Uppsala University, Uppsala, Sweden; 3 Primary Healthcare Center Mora, Region Dalarna, Mora, Sweden; 4 School of Electrical Engineering and Computer Science, KTH Royal Institute of Technology, Stockholm, Sweden; 5 Certec, Department of Design Sciences, Lund University, Lund, Sweden; 6 Division of Family Medicine and Primary Care, Department of Neurobiology, Care Sciences and Society, Karolinska Institutet, Stockholm, Sweden; 7 Theme Heart & Vascular and Neuro, Karolinska University Hospital, Stockholm, Sweden; 8 School of Health and Welfare, Dalarna University, Falun, Sweden

**Keywords:** eHealth, impairment, accessibility, digital inclusion, universal design, disability, digital divide, electronic health, COVID-19, pandemic, cross-sectional study, Sweden, online booking, digital identification, web portal, health information, control group, public health, digital health, digital literacy, health informatics, mobile phone

## Abstract

**Background:**

The adoption of eHealth accelerated during the COVID-19 pandemic. Inequalities in the adoption of eHealth during the COVID-19 pandemic have been reported, but there are few such studies among people with impairment.

**Objectives:**

This study aimed to investigate self-reported use and difficulty in the use of eHealth before the COVID-19 pandemic compared to during late social distancing restrictions in Sweden, among people with and without impairment, as well as between different types of impairment.

**Methods:**

A cross-sectional survey was distributed twice by snowball sampling to people with self-reported impairment and a general population matched by age, gender, and county. Use and difficulty in the use of six eHealth services were compared between groups using chi-square test and logistic regression with year interaction terms, reported as odds ratio adjusted (aOR) for gender and age with 95% CI.

**Results:**

The surveys included 1631 (in 2019) and 1410 (in 2021) participants with impairment, and 1084 (in 2019) and 1223 (in 2021) participants without. Participants with impairment, compared to those without impairment, reported less use and more difficulty in booking health care appointments online, digital identification, and the Swedish national web portal for health information and eHealth services (1177.se), both before and during the pandemic (*P*=.003 or lower). Video health care appointments were the exception to this disability digital divide in eHealth as video appointment adoption was the most likely among participants with attention, executive, and memory impairments (interaction term aOR 2.10, 95% CI 1.30‐3.39). Nonuse and difficulty in the use of eHealth were consistently associated with language impairments and intellectual impairments. For example, language impairments were inversely associated with use of the logged-in eHealth services in 1177.se in 2021 (aOR 0.49, 95% CI 0.36‐0.67) and were associated with difficulty in the use of 1177.se in 2019 (aOR 2.24, 95% CI 1.50‐3.36) and the logged-in eHealth services in 1177.se in 2021 (aOR 1.89, 95% CI 1.32‐2.70). Intellectual impairments were inversely associated with the use of the logged-in eHealth services in 1177.se in 2021 (aOR 0.19, 95% CI 0.13‐0.27).

**Conclusions:**

This repeated cross-sectional survey study, including participants with diverse types of impairment and a control group without impairment, reveals persisting disability digital divides, despite an accelerated adoption of eHealth across the pandemic. eHealth services were not accessible to some groups of people who were identified as being at risk of severe disease during the COVID-19 pandemic. This implies that all people could not use eHealth as a measure of infection protection.

## Introduction

Adoption of eHealth accelerated during the COVID-19 pandemic [1]. Before the pandemic, we identified a disability digital divide in eHealth, that is, people with impairment reported less use and more difficulty in the use of eHealth, particularly with some types of impairment (language impairments and intellectual impairments) [2]. Sustainable development [3], legislation, and human rights principles [4] demand accessible eHealth for all. During the pandemic, people with impairment have reported less use of COVID-19 digital services than people without impairment [5], and have reported difficulty in the use of eHealth [6]. Likewise, eHealth websites have failed to pass accessibility assessments [7]. Even though there are multiple studies and even reviews of associations between eHealth and other sociodemographic factors or somatic diseases, there are very few studies of how people with impairment perceive using eHealth. The three survey studies conducted prior to ours did not sample psychiatric or intellectual impairments, and they had no comparison to before the pandemic [5,6,8]. Further, the two of them that investigated the eHealth modality telehealth reported aggregated results of video health care appointments with telephone contacts.

The emergency phase of the COVID-19 pandemic in the spring of 2020 was characterized by uncertainty [9], and exceptional episodes of social distancing restrictions, including rapid digitalization [1]. Reprioritizing was a necessity, in order to provide acute health care in severe cases of COVID-19, triage with health care guidance, and public health assignments. Like the rest of the general population, health care workers were instructed to self-quarantine when having respiratory tract symptoms, which also limited health care availability [10]. To provide health care services in a social distancing manner, health care facilities were reorganized, separating dedicated areas for suspected COVID-19 cases [11] and the population was encouraged to redirect contacts to eHealth instead of face-to-face contacts [1]. Groups at risk for severe COVID-19 were identified and were recommended intensified social distancing restrictions [12], which included people with intellectual impairments, Down’s syndrome, schizophrenia, bipolar disorder, and stroke. The Swedish restrictions were reimposed in a second wave from autumn 2020 to summer 2021 and a third wave from January 2022 to September 2022. This was a time of realization and concern about prolonged postpandemic scenarios [9].

This study aimed to investigate self-reported use and difficulty in the use of eHealth before the COVID-19 pandemic compared to during late social distancing restrictions in Sweden among people with and without impairment, as well as between different types of impairment.

## Methods

### Study Design

This study had a repeated cross-sectional comparative design, using data from the “Swedes with impairment and the internet 2019” (SMFOI19) survey and the “Swedes with impairment and the internet 2021” (SMFOI21) survey, to people with diverse types of impairment and matched controls.

### Study Setting

The publicly financed Swedish national web portal for health information and eHealth services (1177.se) is open to the whole population and is the most commonly used eHealth service in Sweden [13]. To access the personal eHealth services in 1177.se, it is required that the user have digital identification. Digital identification is also needed to access other private eHealth services. In the past decade, private eHealth services that provide video health care appointments have been launched [14]. Subsequently, this has encouraged the public health care regions to also provide video health care appointments.

### Participants

Participants were people with self-reported impairment and people from the general population matched to the sample of people with impairment by gender, age, and county of residence. Impairment status was self-reported by one questionnaire item with 43 checkboxes of diagnoses and activity limitations and a free-text response option for reporting “Other impairment” (Multimedia Appendix 1). Participants who received the survey as matched controls, but responded that they had impairment, were reallocated to be analyzed as participants with impairment.

### Procedures

The first version of this survey was developed in 2017 to mirror two nationwide Swedish surveys [15,16], out of which the latter is connected to Eurostat [17]. In the SMFOI19 survey, questions on eHealth services were added [2], and SMFOI21 also included questions on video health care appointments. SMFOI19 comprised 47 questions and SMFOI21 comprised 43 questions, on information and communication technology, impairments, and background characteristics. The wording of the questionnaires was developed in collaboration with the Begripsam group, all of whom have lived experience of impairment [18]. Several response modalities were provided (web, pen, phone, or on-site interview) and adaptations were provided upon request (sign language, reading support, speech therapist, or pictograms). Snowball sampling was undertaken from June to October 2019 (SMFOI19) and from May to August 2021 (SMFOI21), respectively [19]. The snowball sampling was distributed by disability networks, as well as by the participants themselves. Then, in February 2020 (SMFOI19) and in March 2022 (SMFOI21), the surveys were posted to six randomly selected matched controls per participant with impairment. Addresses were provided by the Swedish state personal address register [20].

### Data Collection

This study used SMFOI19 and SMFOI21 questionnaire items on eHealth, impairments, and background characteristics.

#### Outcomes

Table 1 provides an overview of outcome variables. In summary, outcomes were measured on the use of eHealth services (ie, whether the services had been used and whether avoiding or preferring use) and difficulty in the use of eHealth services (ie, perceived difficulty or ease of use).

**Table 1.** Outcome variables measuring “use of eHealth” and “difficulty in the use of eHealth.”

Outcome variable followed by response options	In the SMFOI19^a^ survey	In the SMFOI21^b^ survey
Use of booking health care appointments online		
Checkbox items		
“I use booking medical appointments online” or “I use booking dental appointments online”	✓	✓
Use of video health care appointments		
Multiple-choice item		
“Yes, I used video health care appointments prior to the COVID-19 pandemic” or “Yes, I used video health care appointments during the COVID-19 pandemic” Comparisons were made respectively with the response option “No, I have not used video health care appointments,” whereas “Do not know” was treated as missing value		✓
Use of private eHealth services (which are services that primarily provide video health care appointments, chat, and drug prescriptions)		
Checkbox items		
Use of five Swedish private eHealth services or “Other private eHealth service”		✓
Use of the Swedish national web portal for health information and eHealth services (1177.se)		
Checkbox items		
“I use the health information website in the 1177.se”		✓
“I use the logged-in eHealth services in the 1177.se”		✓
Use of digital identification		
Checkbox items		
“I use the digital identification app BankID” or “I use other digital identification than BankID”	✓	✓
Avoid booking health care appointments online		
Multiple-choice item		
“If possible, I avoid booking medical appointments online” or “If possible, I avoid booking dental appointments online” Comparison was made with the response option “I try to book all appointments online,” whereas “Not applicable” was treated as missing value.	✓	✓
Avoid video health care appointments		
Multiple-choice item		
“If possible, I avoid video health care appointments” Comparison was made with the response option “I try to get all my appointments as video health care appointments,” whereas “Not applicable” was treated as missing value.		✓
Difficulty in the use of the Swedish national web portal for health information and eHealth services (1177.se)		
Multiple-choice items		
Two items in SMFOI21 “The health information website in 1177.se is difficult to use” or		✓
“The logged-in eHealth services in 1177.se is difficult to use.” For comparison between SMFOI19 and SMFOI21, they were combined.		✓
One single item in SMFOI19 “The Swedish national web portal for health information and eHealth services (1177.se) is difficult to use” Comparison was made with the response option “It is easy to use,” whereas “Not applicable” was treated as missing value.	✓	
Difficulty in the use of digital identification		
Multiple-choice item		
“It is difficult to use the digital identification app Mobile BankID” or “It is difficult to use other digital identification than BankID” Comparison was made with the response option “It is easy to use,” whereas “Not applicable” was treated as missing value.	✓	✓

a SMFOI19: Swedes with impairment and the internet 2019.

b SMFOI21: Swedes with impairment and the internet 2021.

Data on the use of eHealth were collected in both SMFOI19 and SMFOI21 on booking health care appointments online and digital identification. In SMFOI21, use of eHealth was also collected on video health care appointments “before the pandemic” and “during the pandemic,” private eHealth services, and the Swedish national web portal for health information and eHealth services (1177.se).

Data on the use of eHealth were also collected by questions on whether the respondents “Avoided” or “Preferred” booking health care appointments online in both SMFOI19 and SMFOI21, and video health care appointments in SMFOI21.

Data on difficulties in the use of eHealth were collected in both SMFOI19 and SMFOI21, on the use of the 1177.se and use of digital identification.

#### Independent Variables

Age and gender were selected to be confounding variables based on empirical studies of known demographic associations with the study outcomes [21,22]. Gender was analyzed as “female” and “male” whereas the response options “Other gender” and “Prefer not to answer” were handled as missing values in the analysis. Age was categorized for analysis into four categories: 16‐29, 30‐44, 45‐69, and 70 years of age and older.

Data on impairment were collected by one question with 43 checkbox multiple response options on activity limitations (eg, difficulties in understanding) and diagnoses (eg, intellectual disability), and a free-text option for “Other impairment” (Multimedia Appendix 1). For the analysis, we applied a conceptual model of purposeful subgrouping of impairments, which we had developed in a previous study [2]. The categorization of types of impairment was based on our competence in human-computer interaction, digital accessibility, and medical science, and with the International Classification of Functioning, Disability, and Health (ICF)-classification as a framework [23], as well as empirical knowledge of comorbidity and functioning [24]. The grouping of impairments was done independently by three of the researchers (LP, SJ, and CG). There was almost complete interrater agreement and consensus in a conceptual model. Our conceptual model comprises 11 types of impairments by merging ICF-Body functions and ICF-Activities, as well as the 7 global and 11 specific ICF-Cognitive functions. The robustness of the conceptual model was assessed in a cluster analysis [25] using SPSS (version 26.0; IBM Corp).

#### Background Characteristics

Data on participant characteristics were collected to describe the study samples in regard to educational level, native language, occupation, income, urban or rural status, accommodation, cohabitant status, professional support in everyday life, and access to digital devices.

### Data Analysis

We compared 11 outcomes between participants with and without impairment, as well as between different types of impairment. Six of the outcomes were compared between the survey years (2021 and 2019; Table 1). The statistical software R (version 4.0.3; R Core Team) was used for the analyses. Pair-wise deletion was applied. The significance level was set to ≤.05. Chi-square test and logistic regression with robust standard errors (Huber-White) adjusted for gender and age were used. Multicollinearity was assessed in relation to a predetermined cut-off. Sensitivity analyses indicated the robustness of the estimates, that is, models were fitted with and without independent variables with wide CIs. Results from the following analyses are visualized in figures and presented as numeric values in Multimedia Appendix 2. Among all participants overall, SMFOI21 was compared to SMFOI19, by logistic regression models, adjusted for types of impairment, in addition to adjusting for confounders. Comparisons between participants with impairment to those without were made by chi-square test. To identify types of impairment associated with nonuse and difficulty in the use of eHealth, year-stratified confounder-adjusted logistic regression was used. Comparisons between types of impairment in SMFOI21 to SMFOI19 were performed by the input of multiplicative interaction terms of types of impairment with survey year [26].

### Ethical Considerations

This study was approved by the Swedish Ethical Review Authority (registration 2022-00184-01). The participants provided informed consent prior to participation. No compensation was provided for participation. This study used anonymized data.

## Results

**Figure 1.**
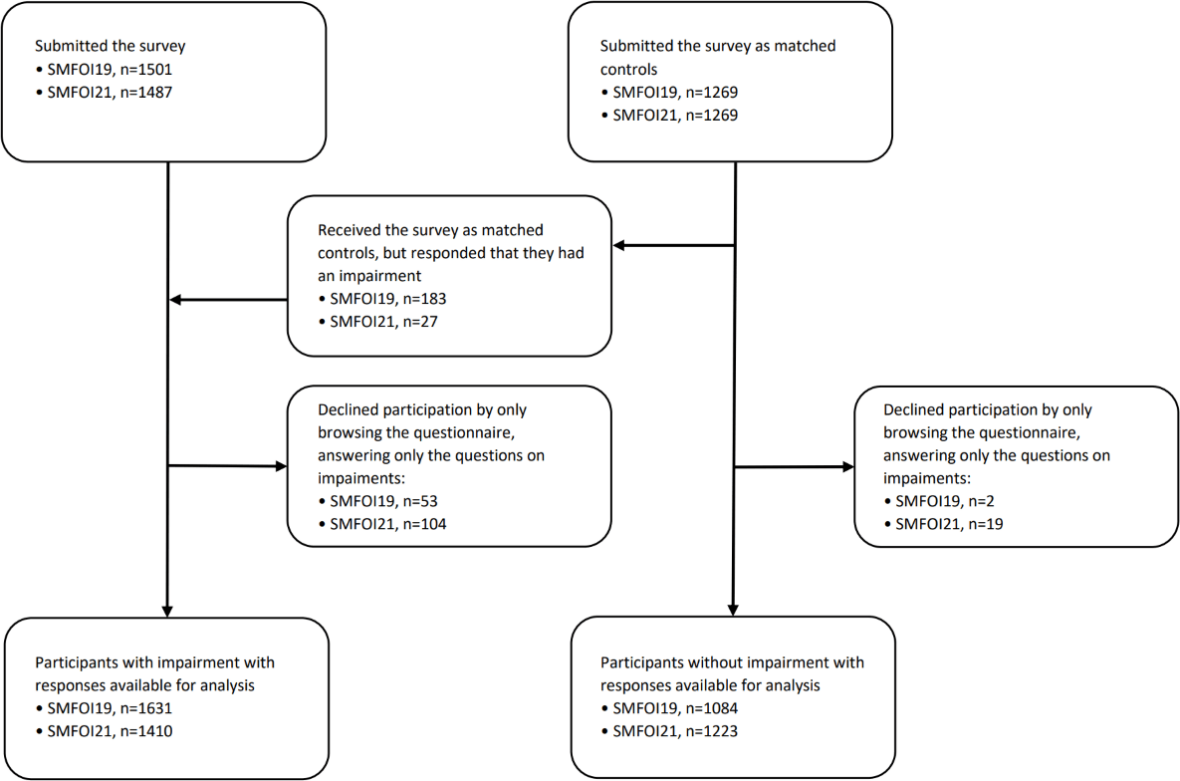
Flowchart of participation in the SMFOI19 and SMFOI21 surveys. SMFOI19: Swedes with impairment and the internet 2019; SMFOI21: Swedes with impairment and the internet 2021.

The flowchart of participation (Figure 1) includes 1631 (in SMFOI19) and 1410 (in SMFOI21) participants with impairment, along with 1084 (in SMFOI19) and 1223 (in SMFOI21) participants without impairment.

Compared to participants without impairment, more of the participants with impairment were educated in a special school, were outside the labor market or not working, had lower income, lived in rental apartments or supported accommodation, were living alone, had professional support in everyday life, and lacked access to digital devices (Table 2).

**Figure 2.**
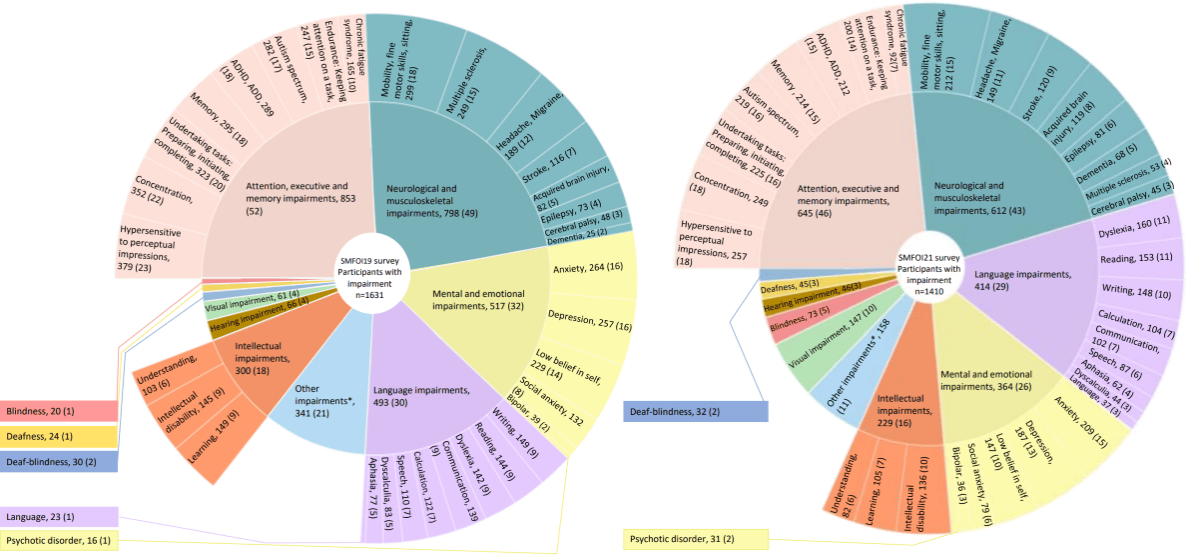
Description of participants’ types of impairment (subgrouped in the inner circle and disaggregated to the level of the questionnaire in the outer circle). The labels are followed by number and proportion of all participants with impairment, n (%). Adds up to over 100% as multiple responses were allowed. The impairment question construction covered both activity limitations (eg, reading difficulties) and diagnoses (eg, dyslexia; Multimedia Appendix 1). *Free-text “other impairments” were in falling frequency: pain, genetic syndromes, paresis, and posttraumatic stress disorder, followed by infrequent responses. ADD: attention deficit disorder; ADHD: attention-deficit/hyperactivity disorder; SMFOI19: Swedes with impairment and the internet 2019; SMFOI21: Swedes with impairment and the internet 2021.

In both survey years, participants with impairment had a median of 2 (IQR 1-6 in SMFOI19 and 1-5 in SMFOI21) impairments. The types of impairment were distributed in approximately similar proportions in the two survey years (Figure 2).

**Table 2.** Background characteristics of participants with and without impairment.

	Participants with impairment in the SMFOI19^a^ survey (n=1631)	Participants without impairment in the SMFOI19 survey (n=1084)	Participants with impairment in the SMFOI21^b^ survey (n=1410)	Participants without impairment in the SMFOI21 survey (n=1223)
Gender, n (%)	n=1356	n=1060	n=1213	n=1193
Women	937 (69)	780 (74)	797 (66)	824 (69)
Men	419 (31)	280 (26)	416 (34)	369 (31)
Age (years), n (%)	n=1388	n=1069	n=1257	n=1200
16‐29	176 (13)	58 (5)	186 (15)	48 (4)
30‐44	304 (22)	156 (15)	269 (21)	173 (14)
45‐69	773 (56)	716 (67)	599 (48)	681 (57)
≥70	135 (10)	139 (13)	203 (16)	298 (25)
Education, n (%)	n=1339	n=1040	n=1222	n=1190
Compulsory school	134 (10)	74 (7)	118 (10)	79 (7)
Special education school	152 (11)	2 (0)	113 (9)	10 (1)
Upper secondary school, Vocational school, or Folk high school (adult education)	567 (42)	442 (43)	488 (40)	447 (38)
University	486 (36)	522 (50)	503 (41)	654 (55)
Native language, n (%)	n=1361	n=1067	n=1253	n=1196
Swedish	1252 (92)	994 (93)	1136 (91)	1065 (89)
Other than Swedish	109 (8)	73 (7)	117 (9)	131 (11)
Occupation, n (%)	n=1367	n=1060	n=1244	n=1194
Retired	273 (17)	304 (28)	327 (26)	478 (40)
Outside of the labor market (disability-related early retirement, daily activity center, or temporary disability allowance)	519 (38)	18 (2)	344 (28)	14 (1)
Working	492 (30)	745 (69)	342 (27)	647 (54)
Student	136 (8)	36 (3)	111 (9)	26 (2)
On the labor market, but not working (unemployed, parental-leave, or sick-leave)	132 (10)	22 (2)	120 (10)	29 (2)
Monthly income (in Swedish Krona, SEK), n (%)	n=1157	n=866	n=1058	n=931
<5000	53 (5)	10 (1)	53 (5)	8 (1)
5000‐24,999	714 (62)	228 (26)	632 (60)	278 (30)
≥25,000	390 (34)	628 (73)	373 (35)	645 (69)
Urban or rural status, n (%)	n=1368	n=1068	n=1239	n=1188
Rural	191 (14)	176 (16)	169 (14)	168 (14)
Suburban or town	325 (24)	252 (24)	284 (23)	290 (24)
Urban	852 (62)	640 (60)	786 (63)	730 (61)
Accommodation, n (%)	n=1365	n=1059	n=1233	n=1194
Supported accommodation (group living, service apartment, or other supported accommodation)	104 (8)	1 (0)	70 (6)	1 (0)
Group living	52 (4)	1 (0)	38 (3)	0 (0)
Service apartment	43 (3)	0 (0)	23 (2)	1 (0)
Other supported accommodation	9 (1)	0 (0)	9 (1)	0 (0)
Apartment, condominium, house, or homeless	1261 (92)	1058 (100)	1163 (94)	1193 (100)
Apartment	470 (34)	168 (16)	407 (33)	225 (19)
Condominium	285 (21)	239 (22)	282 (23)	267 (22)
House	506 (37)	651 (61)	468 (38)	701 (59)
Homeless	0 (0)	0 (0)	6 (0)	0 (0)
Cohabitant status, n (%)	n=1365	n=1066	n=1249	n=1197
Living alone	549 (40)	183 (17)	487 (39)	231 (19)
Cohabiting	816 (60)	883 (83)	762 (61)	966 (81)
Professional support in everyday life, n (%)	n=1381	n=1063	n=1236	n=1190
Have professional support in everyday life	497 (36)	6 (1)	398 (32)	19 (2)
Home based support by municipal care services	112 (8)	2 (0)	91 (7)	4 (0)
Personal assistants	90 (7)	0 (0)	61 (5)	0 (0)
Supported-living staff, support persons or similar	214 (15)	0 (0)	166 (13)	0 (0)
Appointed guardian	111 (8)	0 (0)	71 (6)	0 (0)
Relative	81 (6)	4 (0)	152 (12)	6 (1)
Other support	54 (4)	4 (0)	55 (4)	7 (1)
No support	884 (64)	1057 (99)	838 (68)	1171 (98)
Access to digital devices, n (%)	n=1456	n=1067	n=1316	n=1213
Lack access to at least computer and one portable device	241 (17)	93 (9)	205 (16)	117 (10)
No device	29 (2)	7 (1)	21 (2)	19 (2)
Only computer at home	75 (5)	24 (2)	66 (5)	22 (2)
Only smartphone	78 (5)	19 (2)	44 (3)	30 (2)
Only tablet	17 (1)	1 (0)	13 (1)	1 (0)
Smartphone and tablet	42 (3)	39 (4)	61 (5)	45 (4)
Have access to at least computer and one portable device	1215 (83)	974 (91)	1111 (84)	1096 (90)
Computer and smartphone	418 (29)	264 (25)	395 (30)	362 (30)
Computer and tablet	65 (4)	25 (2)	60 (5)	19 (2)
Computer, smartphone, and tablet	732 (51)	688 (64)	656 (50)	715 (59)
Number of reported impairments	n=1631	n=1084	n=1410	n=1223
Median (IQR), Maximum value	2 (1-6), 20	0 (0-0), 0	2 (1-5), 35	0 (0-0), 0

a SMFOI19: Swedes with impairment and the internet 2019.

b SMFOI21: Swedes with impairment and the internet 2021.

**Figure 3.**
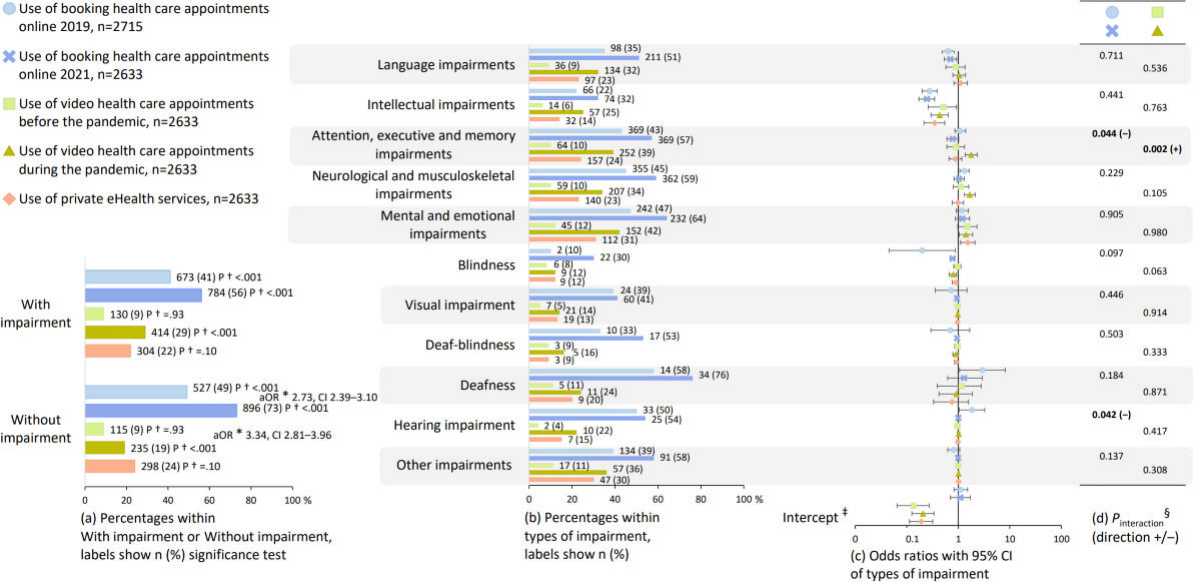
Use of booking health care appointments online, video health care appointments, and private eHealth services. The group with impairment (a) is disaggregated into the subgrouped types of impairment from Figure 2 (b-d). The numeric values visualized in this figure are presented in Tables S1 and S3-S5 in Multimedia Appendix 2. *aOR: adjusted odds ratio from logistic regression comparing use of eHealth among all participants overall between before and during the pandemic (reference group is before the pandemic), adjusted for type of impairment (reference is participants without impairment), gender (reference female), and age (reference <30 years of age); †2-sided *P* value from chi-square test comparing participants with and without impairment. ‡Intercept from year-stratified logistic regression (reference group is participants without impairment), adjusted for gender (reference female), and age (reference <30 years of age). §*P* value from logistic regression interaction terms of year with types of impairment (reference is before the pandemic and participants without impairment), adjusted for gender (reference female), and age (reference <30 years of age).

Comparisons of use between the survey years, as well as between participants with and without impairment, are reported in Figures3a  4a. Among all participants, all eHealth services were used by more participants in SMFOI21 compared to SMFOI19: booking health care appointments online (adjusted odds ratio [aOR] 2.73, 95% CI 2.39‐3.10), video health care appointments (aOR 3.34, 95% CI 2.81‐3.96), and digital identification (aOR 1.74, 95% CI 1.43‐2.10; Figures3a  4a). In both survey years, all but two of the eHealth services were used by fewer participants with impairment compared to participants without impairment: booking health care appointments online (*P*<.001), the information pages in 1177.se (*P*<.001), the logged-in eHealth services in 1177.se (*P*<.001), and digital identification (*P*<.001; Figures3a  4a). The exceptions were video health care appointments, which were used by 9% in both groups (*P*=.93) before the pandemic, and 29% with impairment versus 19% without impairment (*P*<.001) during the pandemic, and private eHealth services (*P*=.10; Figure 3a).

**Figure 4.**
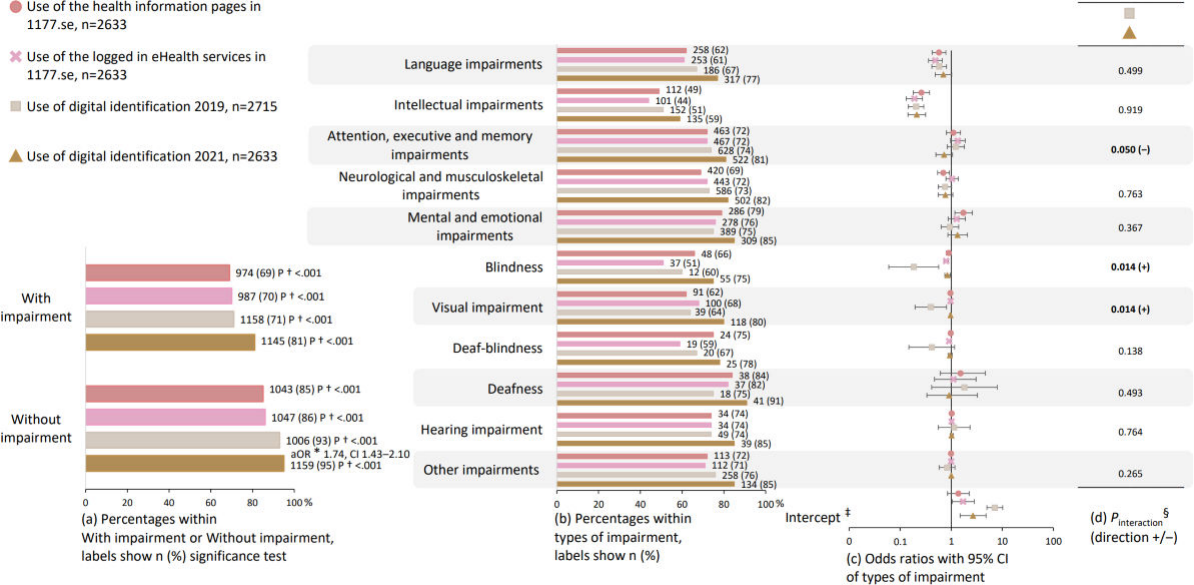
Use of the Swedish national web portal for health information and eHealth services (1177.se) and digital identification. The group with impairment (a) is disaggregated into the subgrouped types of impairment from Figure 2 (b–d). The numeric values visualized in this figure are presented in Tables S1, S6, and S7 in Multimedia Appendix 2. *aOR: adjusted odds ratio from logistic regression comparing use of eHealth among all participants overall between the SMFOI19 and SMFOI21 surveys (reference group is SMFOI19), adjusted for type of impairment (reference is participants without impairment), gender (reference female), and age (reference <30 years of age); †2-sided *P* value from chi-square test comparing participants with and without impairment. ‡Intercept from year-stratified logistic regression (reference group is participants without impairment), adjusted for gender (reference female), and age (reference <30 years of age). §*P* value from logistic regression interaction terms of year with types of impairment (reference is SMFOI19 and participants without impairment), adjusted for gender (reference female), and age (reference <30 years of age). SMFOI19: Swedes with impairment and the internet 2019; SMFOI21: Swedes with impairment and the internet 2021.

Associations between use and types of impairment are reported in Figures3b, c  4b, c, and further, changes in the associations between the survey years are reported in Figures3d  4d. Booking health care appointments online was inversely associated with language impairments in SMFOI19 (aOR 0.64, 95% CI 0.49‐0.83) and in SMFOI21 (aOR 0.70, 95% CI 0.54‐0.93), with intellectual impairments in SMFOI19 (aOR 0.28, 95% CI 0.20‐0.39) and in SMFOI21 (aOR 0.25, 95% CI 0.17‐0.35), and with blindness in SMFOI19 (aOR 0.20, 95% CI 0.05‐0.88) and in SMFOI21 (aOR 0.78, 95% CI 0.70‐0.86; Figure 3c). The use of video health care appointments was inversely associated with intellectual impairments before the pandemic (aOR 0.51, 95% CI 0.26‐0.93) and during the pandemic (aOR 0.44, 95% CI 0.30‐0.64; Figure 3c). Comparing during the pandemic to before, the use of video health care appointments was the most likely to increase among participants with attention, executive, and memory impairments (interaction term aOR 2.10, 95% CI 1.30‐3.39; Figure 3d). The use of private eHealth services was associated with mental and emotional impairments (aOR 1.53, 95% CI 1.11‐2.11) and inversely associated with intellectual impairments (aOR 0.35, 95% CI 0.22‐0.55; Figure 3c). Use of the 1177.se was inversely associated with three types of impairments: language impairments (aOR 0.58, 95% CI 0.43‐0.78 and aOR 0.49, 95% CI 0.36‐0.67), intellectual impairments (aOR 0.26, 95% CI 0.18‐0.38 and aOR 0.19, 95% CI 0.13‐0.27), and blindness (aOR 0.89, 95% CI 0.80‐1.00 and aOR 0.80, 95% 0.72‐0.88; Figure 4c). The use of digital identification was inversely associated with intellectual impairments in SMFOI19 (aOR 0.21, 95% CI 0.15‐0.29), and in SMFOI21 (aOR 0.21, 95% CI 0.14‐0.32), and blindness in SMFOI19 (aOR 0.19, 95% CI 0.06‐0.57) and SMFOI21 (aOR 0.84, 95% CI 0.75‐0.96; Figure 4c).

**Figure 5.**
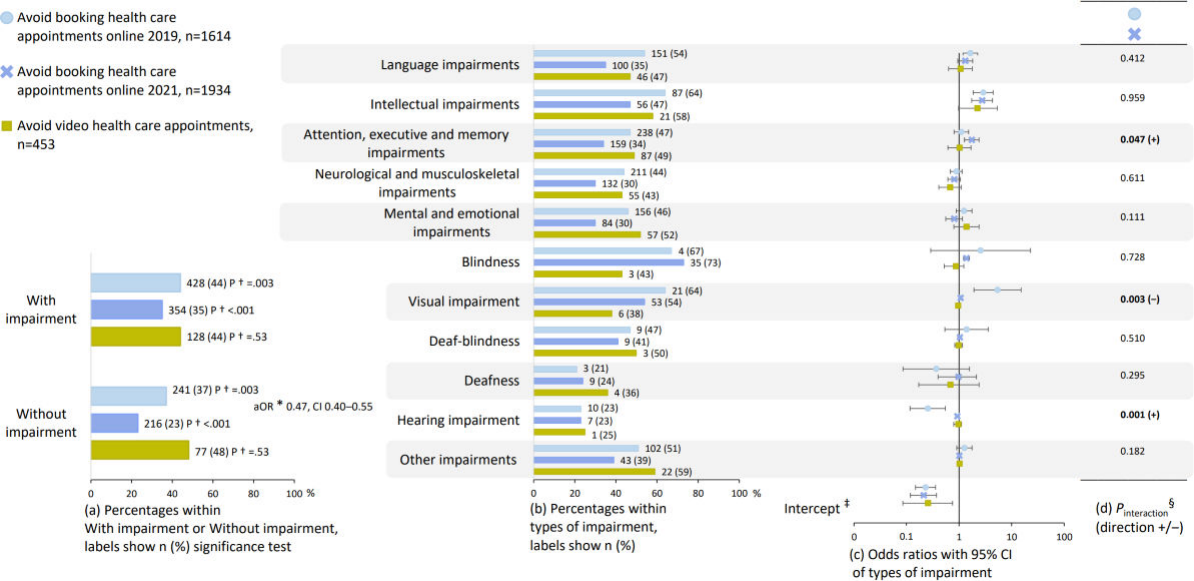
Avoid booking health care appointments online and video health care appointments. The group with impairment (a) is disaggregated into the subgrouped types of impairment from Figure 2 (b-d). The numeric values visualized in this figure are presented in Tables S2, S8, and S9 in Multimedia Appendix 2. *aOR: adjusted odds ratio from logistic regression comparing avoidance of eHealth among all participants overall between the SMFOI19 and SMFOI21 surveys (reference group is 2019), adjusted for type of impairment (reference is participants without impairment), gender (reference female), and age (reference <30 years of age); †2-sided *P* value from chi-square test comparing participants with and without impairment. ‡Intercept from year-stratified logistic regression (reference group is participants without impairment), adjusted for gender (reference female), and age (reference <30 years of age). §*P* value from logistic regression interaction terms of year with types of impairment (reference is SMFOI19 and participants without impairment), adjusted for gender (reference female), and age (reference <30 years of age). SMFOI19: Swedes with impairment and the internet 2019; SMFOI21: Swedes with impairment and the internet 2021.

Comparisons of avoiding use between the survey years, as well as between participants with and without impairment, are reported in Figure 5a. Among all participants, avoiding booking health care appointments online was less frequent in SMFOI21 compared to SMFOI19 (aOR 0.47, 95% CI 0.40‐0.55). Booking health care appointments online was avoided by more participants with impairment compared to participants without impairment in SMFOI19 (44% vs 37%; *P*=.003) and SMFOI21 (35% vs 23%; *P*<.001; Figure 5a). Regarding avoiding video health care appointments, no difference was found between participants with and without impairment (44% vs 48%; *P*=.53; Figure 5).

Associations between avoiding use and types of impairment are reported in Figure 5b,c, and further, changes in the associations between the survey years are reported in Figure 5d. Avoiding booking health care appointments online was associated with intellectual impairments in SMFOI19 (aOR 2.88, 95% CI 1.86‐4.45) and SMFOI21 (aOR 2.74, 95% CI 1.74‐4.33), and visual impairment in SMFOI19 (aOR 5.40, 95% CI 1.92‐15.18) and SMFOI21 (aOR 1.07, 95% CI 1.04‐1.10; Figure 5c). Regarding avoiding video health care appointments, no associations were found with types of impairment (Figure 5c).

**Figure 6.**
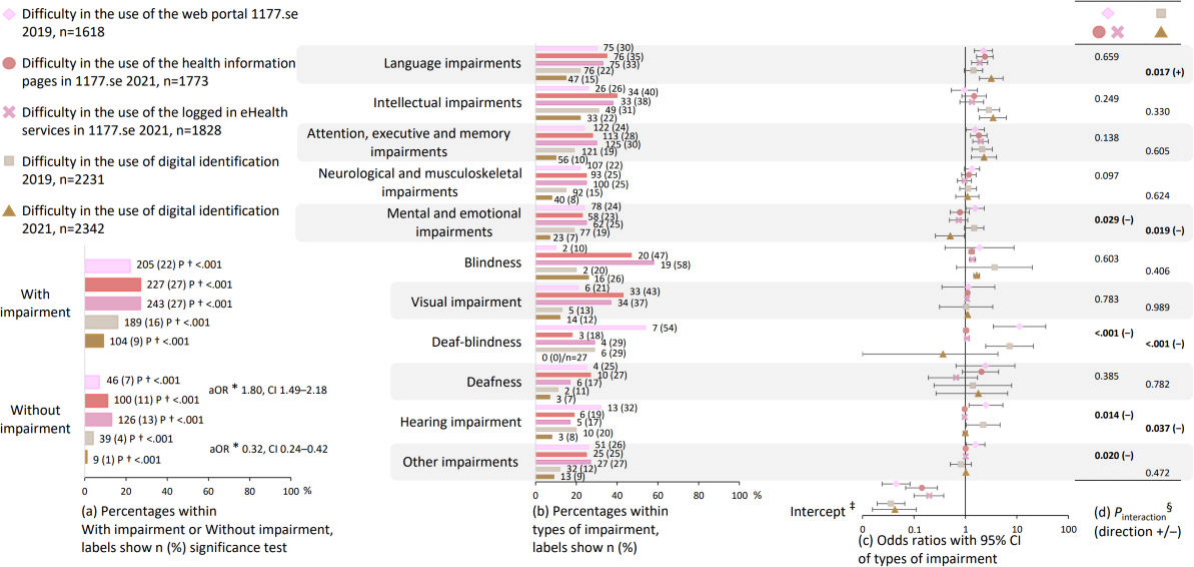
Difficulty in the use of the Swedish national web portal for health information and eHealth services (1177.se) and digital identification. The group with impairment (a) is disaggregated into the subgrouped types of impairment from Figure 2 (b–d). The numeric values visualized in this figure are presented in Tables S2, S10, and S11 in Multimedia Appendix 2. *aOR: adjusted odds ratio from logistic regression comparing difficulty in the use of eHealth among all participants overall between the SMFOI19 and SMFOI21 surveys (reference group is 2019), adjusted for type of impairment (reference is participants without impairment), gender (reference female) and age (reference <30 years of age); †2-sided *P *value from chi-square test comparing participants with and without impairment. ‡Intercept from year-stratified logistic regression (reference group is participants without impairment), adjusted for gender (reference female) and age (reference <30 years of age). §*P* value from logistic regression interaction terms of year with types of impairment (reference is SMFOI19 and participants without impairment), adjusted for gender (reference female) and age (reference <30 years of age). SMFOI19: Swedes with impairment and the internet 2019; SMFOI21: Swedes with impairment and the internet 2021.

Comparisons of difficulty in the use of eHealth services between the survey years, as well as between participants with and without impairment, are reported in Figure 6a. Among all participants, difficulty in the use of the 1177.se was more frequent in SMFOI21 compared to SMFOI19 (aOR 1.80, 95% CI 1.49‐2.18; Figure 6a). Difficulty in the use of digital identification was less frequent in SMFOI21 compared to SMFOI19 (aOR 0.32, 95% CI 0.24‐0.42; Figure 6a). More participants with impairment than participants without impairment reported difficulty in the use of the 1177.se in SMFOI19 (22% vs 7%; *P*<.001), the information pages in 1177.se in SMFOI21 (27% vs 11%; *P*<.001), the logged-in eHealth services in 1177.se in SMFOI21 (27% vs 13%; *P*<.001), and digital identification in SMFOI19 (16% vs 4%; *P*<.001), and in SMFOI21 (9% vs 1%; *P*<.001; Figure 6a).

Associations between difficulty in the use of eHealth services and types of impairment are reported in Figure 6b,c, and further, changes in the associations between the survey years are reported in Figure 6d. Difficulty in the use of the 1177.se was associated with two types of impairments: language impairments and attention, executive, and memory impairments (Figure 6c). Language impairments were associated with difficulty in the use of the 1177.se in SMFOI19 (aOR 2.24, 95% CI 1.50‐3.36), the 1177.se information pages (aOR 2.39, 95% CI 1.65‐3.44), and the logged-in eHealth services (aOR 1.89, 95% CI 1.32‐2.70) in SMFOI21. Attention, executive, and memory impairments were associated with difficulty in the use of the 1177.se in SMFOI19 (aOR 1.55, 95% CI 1.03‐2.34), the 1177.se information pages (aOR 1.82, 95% CI 1.26‐2.62), and the logged-in eHealth services (aOR 1.97, 95% CI 1.39‐2.78) in SMFOI21. Difficulty in the use of digital identification was associated with intellectual impairments in SMFOI19 (aOR 2.86, 95% CI 1.77‐4.62) and in SMFOI21 (aOR 3.44, 95% CI 1.87‐6.26), and attention, executive, and memory impairments in SMFOI19 (aOR 2.11, 95% CI 1.33‐3.34) and in SMFOI21 (aOR 2.30, 95% CI 1.30‐4.02; Figure 6c).

## Discussion

### Principal Findings

In a rapid digitalization in health care, this first comparison across the COVID-19 pandemic revealed disability digital divides in eHealth that remained proportional. In this comparison of people with and without impairment, the former were disfavored in regard to eHealth. Namely, they reported less use of booking health care appointments online, the 1177.se and digital identification, which is in line with most studies before the pandemic as discussed in our previous article [2] and one during the pandemic [5]. Further, they reported substantial difficulty in the use of eHealth, which is in agreement with a survey conducted during the pandemic [6]. Nonuse and difficulty in the use of eHealth were consistently associated with language impairments and intellectual impairments. Our findings show that the highest adoption of video health care appointments was among participants with attention, executive, and memory impairments, which is in line with other surveys during the pandemic [8,27]. The latter of the cited studies concluded that video health care appointments increased among mental health service users when contacts were redirected to eHealth instead of face-to-face [27]. Accordingly, it is plausible that the adoption of video health care appointments among participants with attention, executive, and memory impairments in this study was due to disrupted face-to-face health care provision.

The Swedish national web portal for health information and eHealth services, 1177.se, was more frequently reported as difficult to use in SMFOI21 than in SMFOI19, both among participants with and without impairment, despite an accelerated adoption of eHealth across the pandemic. This may be interpreted as a sign of forced digitalization, which implies that eHealth followed a previously shown societal pattern of nonaccessible social distancing restrictions [28]. People with intellectual impairments were afflicted on two fronts. They were distinguished as a risk group for severe COVID-19 [12] and thereby recommended intensified social distancing restrictions, which meant that they were encouraged to redirect contacts to eHealth instead of face-to-face contacts with health care if possible, while at the same time, they were facing substantial difficulties in the use of eHealth. Cognitive accessibility is insufficiently covered in eHealth studies [29,30] and guidelines [30]. Known adaptations for cognitive accessibility are intuitive structure, important elements before page scroll, large icons, visualizations, and third-party participation [31,32]. However, there is a lack of conformance to what is known about accessibility [7]. Moreover, in this study, people with language impairments were particularly afflicted by difficulties in the use of digital services, which has previously been shown by multiple studies [10,33], but not all [5]. Text and numerical tasks can be adapted by standards for understandable text, autocorrect, data import, speech recognition, audio, and enabling enough duration [31,32]. Barriers to the use of video appointments can be due to reduced nonverbal communication [10] and natural pauses [33].

### Implications

Until this disability digital divide in eHealth is bridged, practitioners should provide eHealth integrated with staff support, or maintain conventional services as alternatives to digitalized options. In line with policy [34], we underscore that research and development must conform to legislated accessibility standards [4,31,32,35]. But in addition, we suggest that user participation in the design of eHealth [36] and adherence to principles for universal design [37] can bring additional benefits, beyond the accessibility standards. Such benefits exceed accessibility, by, for instance, readability, clarity, interoperability, and assistive functions, which improve usability for all users [32,35].

### Strengths and Limitations

This is, to our knowledge, the first study comparing the accessibility of eHealth across the COVID-19 pandemic among people with diverse types of impairment relevant to the risk of disablement by poorly designed digital services. The pandemic provoked exceptional conditions, which can provide insights for developing resilient health care systems, in which people with high health care needs are included. This comparison between two cross-sectional surveys was deemed a suitable study design to study an unanticipated historical event, in the absence of an available cohort study.

Snowball sampling [19] is a major reason for achieving representation of seldom-heard populations. However, it is plausible that this sampling method engaged more people with higher digital literacy to respond to the survey. Further, self-assessment of digital literacy can be associated with overestimation of skills [38]. Thus, in summation, while this study did demonstrate the disability digital divide in eHealth, it may have underestimated its magnitude. In the two study samples, participants’ characteristics and types of impairment demonstrate similar proportions, which makes it plausible that misclassification did not obstruct drawing inferences on comparisons between samples. To allow representation of individuals with multimorbidity in the analysis, we assessed that multicollinearity did not exceed the predetermined cut-off, and that outcomes were not associated with the number of reported impairments. The SMFOI21 survey sampled more participants in the oldest group (70 years or older) and less participants of 45‐69 years of age, as compared to the SMFOI19 survey. This was handled as we adjusted for age in the statistical analysis. While we adjusted for gender and age, there were socioeconomic differences between people with and without impairment, for which we did not control. The dependent variables were investigated by different types of response options “I use”; “I avoid” or “I prefer”; as well as “is difficult to use” or “is easy to use” in order to capture different aspects of the phenomenon. We consider that avoiding is a dimension of using, in that it measures a type of “not using.” However, it is possible that avoidance also can be associated with difficulty of use, but may also be influenced by other mechanisms, for example, self-efficacy, normative beliefs, or social support. This study focused on the association between types of impairment and eHealth services for patient-provider contact, meaning that other types of eHealth and other factors plausibly mediating the use of eHealth were not investigated, that is, eHealth literacy, socioeconomic factors, or lacking access to digital devices.

### Conclusions

Despite an accelerated adoption of eHealth across the COVID-19 pandemic, our results show no relevant difference in the disability digital divide in eHealth between the survey years. In both before and during the pandemic, there were substantial nonuse and difficulty in the use of eHealth among people with impairment. Nonuse and difficulty in the use of eHealth were consistently associated with language impairments and intellectual impairments. This implies that groups recognized as being at risk of severe COVID-19 could not use eHealth, while emphasis was put on eHealth as a measure of infection protection in the social distancing policy. Accessibility standards can improve services. Until then, our results indicate that eHealth in its current state should be provided with optional support, or only be provided as a complement to conventional contacts.

## Supplementary material

10.2196/64707 Multimedia Appendix 1 Question about impairments.

10.2196/64707 Multimedia Appendix 2 Tables of all logistic regression models of this study. Unadjusted and adjusted model per outcome.

## Abbreviations

aOR: adjusted odds ratio
ICF: International Classification of Functioning, Disability, and Health
SMFOI19: Swedes with impairment and the internet 2019
SMFOI21: Swedes with impairment and the internet 2021
