# Supplementary material for: Accessibility of eHealth Before and During the COVID-19 Pandemic Among People With and People Without Impairment: Repeated Cross-Sectional Survey
Source: JMIR Public Health Surveill. 2025 Mar 28;11:e64707. doi: 10.2196/64707 (PMC11999378; doi:10.2196/64707)
Supplement: Multimedia Appendix 1 [file publichealth-v11-e64707-s001.docx]

## Which one(s) of these diagnoses, impairments and/or difficulties do you have?

Go through the checklist and tic the boxes that apply to you. You can select several options. You can also tic the box ‘Other’ at the bottom of the list and specify other diagnoses, impairments or difficulties that are relevant to you.

- ADD
- ADHD
- Aphasia
- Autism Spectrum (Autism, Asperger Syndrome)
- Bipolar disorder
- Blindness
- Cerebral Palsy / CP
- Dementia, Alzheimer etc.
- Depression
- Dyslexia
- Dyscalculia
- Deafness, childhood onset
- Deafness, acquired in adulthood
- Deaf blindness
- Epilepsy
- Severe hearing impairment
- Severe visual impairment
- Acquired brain injury
- Headache, Migraine
- Communication difficulties
- Concentration difficulties
- Chronic Fatigue Syndrome / ME
- Hypersensitive to strong/sudden perceptual impressions (flickering lights, sudden or loud sounds, etc.)
- Reading difficulties
- Memory difficulties
- Multiple Sclerosis / MS
- Parkinson Disease
- Mathematical difficulties
- Mobility impairment, difficulties in fine motor skills
- Stroke
- Schizophrenia, psychotic disorder
- Low self-esteem / low belief in self
- Writing difficulties
- Social anxiety
- Language disorder, DLD
- Difficulties understanding
- Difficulties learning new things
- Difficulties getting started, or completing, tasks or activities
- Difficulties to sit
- Difficulties keeping attention on a task or activity
- Speech difficulties
- Intellectual disability
- Anxiety
- Other, please describe:
