# Supplementary material for: Accessibility of eHealth Before and During the COVID-19 Pandemic Among People With and People Without Impairment: Repeated Cross-Sectional Survey
Source: JMIR Public Health Surveill. 2025 Mar 28;11:e64707. doi: 10.2196/64707 (PMC11999378; doi:10.2196/64707)
Supplement: Multimedia Appendix 2 [file publichealth-v11-e64707-s002.docx]

## Table S1. Logistic regression models comparing use of eHealth among all participants overall, between before and during the pandemic

|  | **Use of booking health care appointments online** | | | **Use of video health care appointments** | | | **Use of digital identification** | | |
| --- | --- | --- | --- | --- | --- | --- | --- | --- | --- |
|  |  | n=5348 | n=4813 |  | n=2633 | n=2404 |  | n=5348 | n=4813 |
| **Independent variables** | n(%) | OR(*P*) CI | aOR(*P*) CI | n(%) | OR(*P*) CI | aOR(*P*) CI | n(%) | OR(*P*) CI | aOR(*P*) CI |
| Among all participants overall, comparison between before and during the pandemic | 1680(64) | 2·23**(<0·001)** 1·99–2·48 | 2·73**(<0·001)** 2·39–3·10 | 649(25) | 3·19**(<0·001)** 2·72–3·74 | 3·34**(<0·001)** 2·81–3·96 | 2304(88) | 1·78**(<0·001)** 1·54–2·07 | 1·74**(<0·001)** 1·43–2·10 |
| Intercept* | 1200(44) | 0·79**(<0·001)** 0·73–0·85 | 0·40**(<0·001)** 0·29–0·55 | 245(9) | 0·10**(<0·001)** 0·09–0·12 | 0·19**(<0·001)** 0·12–0·29 | 2164(80) | 3·93**(<0·001)** 3·58–4·31 | 1·36**(0·133)** 0·91–2·03 |

* Reference group is 2019 results, adjusted for type of impairment (reference is participants without impairment), gender (reference female) and age (reference <30 years of age); aOR = adjusted odds ratio; *P* = *P*-value; CI = 95% confidence interval.

## Table S2. Logistic regression models comparing avoiding/difficulty in the use of eHealth among all participants overall, between the SMFOI19 and SMFOI21 surveys

|  | **Avoid booking health care appointments online** | | | **Difficulty in the use of the Swedish national web portal for health information and eHealth services (1177.se)** | | | **Difficulty in the use of digital identification** | | |
| --- | --- | --- | --- | --- | --- | --- | --- | --- | --- |
|  |  | n=3548 | n=3309 |  | n=3677 | n=3485 |  | n=4573 | n=4283 |
| **Independent variables** | n(%) | OR(*P*) CI | aOR(*P*) CI | n(%) | OR(*P*) CI | aOR(*P*) CI | n(%) | OR(*P*) CI | aOR(*P*) CI |
| Among all participants overall, comparison between the SMFOI19 and SMFOI21 surveys | 570(29) | 0·59**(<0·001)** 0·51–0·68 | 0·47**(<0·001)** 0·40–0·55 | 519(25) | 1·84**(<0·001)** 1·55–2·17 | 1·80**(<0·001)** 1·49–2·18 | 113(5) | 0·45**(<0·001)** 0·35–0·56 | 0·32**(<0·001)** 0·24–0·42 |
| Intercept* | 669(41) | 0·71**(<0·001)** 0·64–0·78 | 0·44**(<0·001)** 0·29–0·68 | 251(16) | 0·18**(<0·001)** 0·16–0·21 | 0·11**(<0·001)** 0·07–0·18 | 228(10) | 0·11**(<0·001)** 0·10–0·13 | 0·10**(<0·001)** 0·05–0·17 |

* Reference group is 2019 results, adjusted for type of impairment (reference is participants without impairment), gender (reference female) and age (reference <30 years of age); aOR = adjusted odds ratio; *P* = *P*-value; CI = 95% confidence interval.

## Table S3. Logistic regression models of booking health care appointments online, depending on types of impairment, year-stratified as well comparison between the SMFOI19 and SMFOI21 surveys

|  | **Use of booking health care appointments online 2019** | | | **Use of booking health care appointments online 2021** | | | | **Comparison between SMFOI21 and SMFOI19 Interaction term model** | | | |  |
| --- | --- | --- | --- | --- | --- | --- | --- | --- | --- | --- | --- | --- |
|  |  | n=2715 | n=2409 |  | n=2633 | n=2404 | |  | |  | |  |
| **Independent variables** | n(%) | OR(*P*) CI | aOR(*P*) CI | n(%) | OR(*P*) CI | aOR(*P*) CI | | OR(*P*) CI | | aOR(*P*) CI | |  |
| Language impairments | 98(35) | 0·71**(0·003)** 0·56–0·89 | 0·64**(0·001)** 0·49–0·83 | 211(51) | 0·69**(0·004)** 0·54–0·89 | | 0·70**(0·012)** 0·54–0·93 | | 0·98(0·907) 0·69–1·39 | | 1·08(0·711) 0·73–1·59 | |
| Intellectual impairments | 66(22) | 0·31**(<0·001)** 0·23–0·42 | 0·28**(<0·001)** 0·20–0·39 | 74(32) | 0·26**(<0·001)** 0·19–0·36 | | 0·25**(<0·001)** 0·17–0·35 | | 0·83(0·409) 0·53–1·29 | | 0·82(0·441) 0·50–1·35 | |
| Attention, executive and memory impairments | 369(43) | 1·07(0·52) 0·87–1·32 | 1·10(0·45) 0·86–1·40 | 369(57) | 0·89(0·343) 0·70–1·13 | | 0·78(0·074) 0·60–1·02 | | 0·83(0·263) 0·60–1·15 | | 0·69**(0·044)** 0·48–0·99 | |
| Neurological and musculoskeletal impairments | 355(45) | 1·18(0·08) 0·98–1·41 | 1·32**(0·008)** 1·08–1·63 | 362(59) | 0·99(0·960) 0·81–1·23 | | 1·04(0·755) 0·82–1·31 | | 0·84(0·243) 0·63–1·12 | | 0·82(0·229) 0·60–1·13 | |
| Mental and emotional impairments | 242(47) | 1·43**(0·004)** 1·12–1·81 | 1·20(0·20) 0·91–1·57 | 232(64) | 1·43**(0·012)** 1·08–1·90 | | 1·20(0·254) 0·88–1·65 | | 1·00(0·985) 0·69–1·47 | | 0·97(0·905) 0·64–1·49 | |
| **Sensory impairments** |  |  |  |  |  | |  | |  | |  | |
| Blindness | 2(10) | 0·12**(0·005)** 0·027–0·53 | 0·20**(0·03)** 0·046–0·88 | 22(30) | 0·76**(<0·001)** 0·69–0·82 | | 0·78**(<0·001)** 0·70–0·86 | | 6·38**(0·021)** 1·33–30·69 | | 3·93(0·097) 0·78–19·74 | |
| Visual impairment | 24(39) | 0·77(0·36) 0·44–1·34 | 0·73(0·38) 0·36–1·48 | 60(41) | 0·93**(<0·001)** 0·91–0·95 | | 0·94**(<0·001)** 0·92–0·97 | | 1·21(0·506) 0·69–2·14 | | 1·33(0·446) 0·64–2·74 | |
| Deaf-blindness | 10(33) | 0·61(0·21) 0·28–1·33 | 0·70(0·42) 0·29–1·67 | 17(53) | 0·95(0·064) 0·90–1·00 | | 0·95(0·145) 0·89–1·02 | | 1·56(0·281) 0·69–3·52 | | 1·37(0·503) 0·55–3·43 | |
| Deafness | 14(58) | 1·81(0·18) 0·76–4·35 | 2·95**(0·04)** 1·06–8·22 | 34(76) | 1·56(0·217) 0·80–3·33 | | 1·29(0·512) 0·62–2·92 | | 0·86(0·800) 0·27–2·72 | | 0·42(0·184) 0·12–1·51 | |
| Hearing impairment | 33(50) | 1·36(0·22) 0·83–2·24 | 1·85**(0·03)** 1·05–3·27 | 25(54) | 1·00(0·852) 0·95–1·04 | | 1·01(0·762) 0·96–1·06 | | 0·73(0·229) 0·44–1·22 | | 0·54**(0·042)** 0·30–0·98 | |
| Other impairments | 134(39) | 0·80(0·06) 0·63–1·01 | 0·82(0·15) 0·62–1·07 | 91(58) | 0·99(0·280) 0·99–1·00 | | 1·00(0·433) 0·99–1·01 | | 1·25(0·070) 0·98–1·60 | | 1·23(0·137) 0·94–1·62 | |
| Intercept * | 527(49) | 0·85**(0·003)** 0·77–0·95 | 1·12 (0·46) 0·83–1·52 | 896(73) | 2·37**(<0·001)** 2·13–2·65 | | 1·09(0·688) 0·70–1·71 | | 0·85**(0·003)** 0·77–0·95 | | 0·39**(<0·001)** 0·28–0·54 | |

* Reference group is participants without impairment, adjusted for gender (reference female) and age (reference <30 years of age). Further, for the interaction term models, reference is 2019 results as interaction terms with types of impairment. aOR = adjusted odds ratio; *P* = *P*-value; CI = 95% confidence interval.

## Table S4. Logistic regression models of use of video health care appointments, depending on types of impairment, year-stratified as well as comparison between before and during the pandemic

|  | **Use of video health care appointments before the pandemic** | | | **Use of video health care appointments during the pandemic** | | | **Before vs during the pandemic Interaction term model** | | |
| --- | --- | --- | --- | --- | --- | --- | --- | --- | --- |
|  |  | n=2633 | n=2404 |  | n=2633 | n=2404 |  |  | |
| **Independent variables** | n(%) | OR(*P*) CI | aOR(*P*) CI | n(%) | OR(*P*) CI | aOR(*P*) CI | OR(*P*) CI | | aOR(*P*) CI |
| Language impairments | 36(9) | 0·92(0·685) 0·60–1·37 | 0·89(0·606) 0·58–1·36 | 134(32) | 1·09(0·513) 0·84–1·42 | 1·05(0·759) 0·78–1·39 | 1·19(0·498) 0·72–1·97 | 1·18(0·536) 0·70–1·98 | |
| Intellectual impairments | 14(6) | 0·56(0·059) 0·30–0·99 | 0·51**(0·036)** 0·26–0·93 | 57(25) | 0·50**(<0·001)** 0·35–0·71 | 0·44**(<0·001)** 0·30–0·64 | 0·90(0·756) 0·45–1·80 | 0·90(0·763) 0·44–1·83 | |
| Attention, executive and memory impairments | 64(10) | 1·01(0·951) 0·69–1·47 | 0·90(0·604) 0·60–1·33 | 252(39) | 2·11**(<0·001)** 1·66–2·69 | 1·78**(<0·001)** 1·37–2·32 | 2·09**(0·001)** 1·33–3·28 | 2·10**(0·002)** 1·30–3·39 | |
| Neurological and musculoskeletal impairments | 59(10) | 1·06(0·749) 0·75–1·46 | 1·13(0·481) 0·80–1·59 | 207(34) | 1·44**(0·001)** 1·16–1·79 | 1·68**(<0·001)** 1·32–2·13 | 1·36(0·127) 0·92–2·03 | 1·41(0·105) 0·93–2·15 | |
| Mental and emotional impairments | 45(12) | 1·52**(0·047)** 1·00–2·28 | 1·51(0·062) 0·97–2·31 | 152(42) | 1·57**(0·001)** 1·19–2·05 | 1·41**(0·025)** 1·04–1·91 | 1·03(0·899) 0·63–1·69 | 1·01(0·980) 0·59–1·72 | |
| **Sensory impairments** |  |  |  |  |  |  |  |  | |
| Blindness | 6(8) | 0·97(0·622) 0·82–1·10 | 0·98(0·776) 0·82–1·13 | 9(12) | 0·84**(0·006)** 0·74–0·94 | 0·80**(0·007)** 0·67–0·93 | 0·87(0·167) 0·72–1·06 | 0·80(0·063) 0·64–1·01 | |
| Visual impairment | 7(5) | 0·96(0·078) 0·91–1·00 | 0·97(0·231) 0·92–1·01 | 21(14) | 0·96**(0·006)** 0·93–0·99 | 0·98(0·183) 0·95–1·01 | 1·00(0·976) 0·95–1·06 | 1·00(0·914) 0·95–1·06 | |
| Deaf-blindness | 3(9) | 1·00(0·953) 0·89–1·08 | 0·99(0·810) 0·85–1·09 | 5(16) | 0·96(0·339) 0·89–1·03 | 0·90(0·08) 0·78–1·00 | 0·97(0·595) 0·85–1·09 | 0·92(0·333) 0·77–1·09 | |
| Deafness | 5(11) | 1·19(0·724) 0·40–2·78 | 1·16(0·766) 0·39–2·78 | 11(24) | 1·06(0·867) 0·50–2·08 | 0·92(0·823) 0·42–1·87 | 0·90(0·855) 0·28–2·91 | 0·91(0·871) 0·27–2·99 | |
| Hearing impairment | 2(4) | 0·96(0·364) 0·85–1·04 | 0·97(0·560) 0·86–1·05 | 10(22) | 1·00(0·917) 0·95–1·05 | 1·02(0·437) 0·97–1·07 | 1·04(0·463) 0·93–1·17 | 1·05(0·417) 0·94–1·17 | |
| Other impairments | 17(11) | 1·00(0·545) 0·99–1·02 | 1·00(0·639) 0·99–1·02 | 57(36) | 1·01**(0·03)** 1·00–1·02 | 1·01**(0·013)** 1·00–1·02 | 1·01(0·478) 0·99–1·02 | 1·01(0·308) 0·99–1·03 | |
| Intercept * | 115(9) | 0·10**(<0·001)** 0·09–0·12 | 0·14**(<0·001)** 0·07–0·27 | 235(19) | 0·24**(<0·001)** 0·21–0·27 | 0·21**(<0·001)** 0·13–0·34 | 0·10**(<0·001)** 0·09–0·12 | 0·10**(<0·001)** 0·07–0·16 | |

* Reference group is participants without impairment, adjusted for gender (reference female) and age (reference <30 years of age). Further, for the interaction term models, reference is ‘before the pandemic’ as interaction terms with types of impairment. aOR = adjusted odds ratio; *P* = *P*-value; CI = 95% confidence interval.

## Table S5. Logistic regression models of use of private eHealth services, depending on types of impairment (investigated only in the SMFOI21 survey)

|  | **Use of private eHealth services** | | |
| --- | --- | --- | --- |
|  |  | n=2633 | n=2404 |
| **Independent variables** | n(%) | OR(*P*) CI | aOR(*P*) CI |
| Language impairments | 97(23) | 1·09(0·530) 0·82–1·44 | 1·11(0·482) 0·82–1·50 |
| Intellectual impairments | 32(14) | 0·43**(<0·001)** 0·28–0·65 | 0·35**(<0·001)** 0·22–0·55 |
| Attention, executive and memory impairments | 157(24) | 0·98(0·865) 0·75–1·27 | 0·88(0·391) 0·66–1·17 |
| Neurological and musculoskeletal impairments | 140(23) | 0·97(0·828) 0·77–1·23 | 0·99(0·934) 0·77–1·27 |
| Mental and emotional impairments | 112(31) | 1·71**(<0·001)** 1·27–2·28 | 1·53**(0·009)** 1·11–2·11 |
| **Sensory impairments** |  |  |  |
| Blindness | 9(12) | 0·87**(0·02)** 0·76–0·97 | 0·89(0·09) 0·76–1·01 |
| Visual impairment | 19(13) | 0·96**(0·005)** 0·93–0·98 | 0·97(0·058) 0·94–1·00 |
| Deaf-blindness | 3(9) | 0·92(0·059) 0·82–0·99 | 0·93(0·148) 0·83–1·01 |
| Deafness | 9(20) | 0·80(0·563) 0·36–1·62 | 0·76(0·481) 0·33–1·57 |
| Hearing impairment | 7(15) | 0·98(0·377) 0·92–1·03 | 0·99(0·794) 0·93–1·05 |
| Other impairments | 47(30) | 1·01**(0·034)** 1·00–1·02 | 1·01**(0·02)** 1·00–1·02 |
| Intercept * | 298(24) | 0·30**(<0·001)** 0·27–0·34 | 0·19**(<0·001)** 0·11–0·32 |

* Reference group is participants without impairment, adjusted for gender (reference female) and age (reference <30 years of age). aOR = adjusted odds ratio; *P* = *P*-value; CI = 95% confidence interval.

## Table S6. Logistic regression models of use of the Swedish national web portal for health information and eHealth services (1177.se), depending on types of impairment (investigated only in the SMFOI21 survey)

|  | **Use of the health information pages in the Swedish national web portal for health information and eHealth services (1177.se)** | | | **Use of the logged-in eHealth services in the Swedish national web portal for health information and eHealth services (1177.se)** | | |
| --- | --- | --- | --- | --- | --- | --- |
|  |  | n=2633 | n=2404 |  | n=2633 | n=2404 |
| **Independent variables** | n(%) | OR(*P*) CI | aOR(*P*) CI | n(%) | OR(*P*) CI | aOR(*P*) CI |
| Language impairments | 258(62) | 0·56**(<0·001)** 0·43–0·73 | 0·58**(<0·001)** 0·43–0·78 | 253(61) | 0·48**(<0·001)** 0·37–0·64 | 0·49**(<0·001)** 0·36–0·67 |
| Intellectual impairments | 112(49) | 0·30**(<0·001)** 0·22–0·41 | 0·26**(<0·001)** 0·18–0·38 | 101(44) | 0·21**(<0·001)** 0·15–0·29 | 0·19**(<0·001)** 0·13–0·27 |
| Attention, executive and memory impairments | 463(72) | 1·06(0·684) 0·81–1·39 | 1·10(0·544) 0·81–1·52 | 467(72) | 1·21(0·180) 0·92–1·61 | 1·34(0·08) 0·97–1·88 |
| Neurological and musculoskeletal impairments | 420(69) | 0·71**(0·003)** 0·57–0·89 | 0·70**(0·009)** 0·54–0·92 | 443(72) | 0·98(0·868) 0·77–1·25 | 1·04(0·796) 0·79–1·38 |
| Mental and emotional impairments | 286(79) | 1·80**(<0·001)** 1·30–2·50 | 1·73**(0·006)** 1·18–2·59 | 278(76) | 1·39**(0·046)** 1·01–1·93 | 1·28(0·212) 0·87–1·89 |
| **Sensory impairments** |  |  |  |  |  |  |
| Blindness | 48(66) | 0·88**(0·003)** 0·81–0·96 | 0·89**(0·04)** 0·80–1·00 | 37(51) | 0·77**(<0·001)** 0·71–0·84 | 0·80**(<0·001)** 0·72–0·88 |
| Visual impairment | 91(62) | 0·95**(<0·001)** 0·93–0·97 | 0·96**(0·003)** 0·94–0·99 | 100(68) | 0·96**(0·002)** 0·94–0·99 | 0·97(0·066) 0·95–1·00 |
| Deaf-blindness | 24(75) | 0·98(0·472) 0·92–1·05 | 0·97(0·537) 0·90–1·07 | 19(59) | 0·92**(0·003)** 0·87–0·97 | 0·91**(0·007)** 0·85–0·98 |
| Deafness | 38(84) | 1·47(0·367) 0·68–3·67 | 1·52(0·407) 0·61–4·66 | 37(82) | 1·12(0·773) 0·54–2·67 | 1·10(0·836) 0·47–3·07 |
| Hearing impairment | 34(74) | 1·02(0·506) 0·97–1·07 | 1·02(0·457) 0·97–1·08 | 34(74) | 1·01(0·788) 0·96–1·06 | 1·02(0·490) 0·97–1·08 |
| Other impairments | 113(72) | 0·99(0·265) 0·98–1·00 | 1·00(0·463) 0·98–1·01 | 112(71) | 0·99(0·126) 0·98–1·00 | 0·99(0·317) 0·98–1·01 |
| Intercept * | 1043(85) | 4·53**(<0·001)** 3·99–5·15 | 1·38(0·199) 0·85–2·25 | 1047(86) | 4·84**(<0·001)** 4·26–5·52 | 1·71**(0·035)** 1·04–2·83 |

* Reference group is participants without impairment, adjusted for gender (reference female) and age (reference <30 years of age). aOR = adjusted odds ratio; *P* = *P*-value; CI = 95% confidence interval.

## Table S7. Logistic regression models of use of digital identification, depending on types of impairment, year-stratified as well as comparison between the SMFOI19 and SMFOI21 surveys

|  | **Use of digital identification 2019** | | | **Use of digital identification 2021** | | | **SMFOI21 vs SMFOI19 Interaction term model** | |
| --- | --- | --- | --- | --- | --- | --- | --- | --- |
|  |  | n=2715 | n=2409 |  | n=2633 | n=2404 |  |  |
| **Independent variables** | n(%) | OR(*P*) CI | aOR(*P*) CI | n(%) | OR(*P*) CI | aOR(*P*) CI | OR(*P*) CI | aOR(*P*) CI |
| Language impairments | 186(67) | 0·66**(0·003)** 0·50–0·87 | 0·58**(0·001)** 0·42–0·81 | 317(77) | 0·67**(0·016)** 0·48–0·93 | 0·70(0·059) 0·49–1·02 | 1·01(0·963) 0·64–1·59 | 1·20(0·499) 0·71–2·01 |
| Intellectual impairments | 152(51) | 0·25**(<0·001)** 0·19–0·34 | 0·21**(<0·001)** 0·15–0·29 | 135(59) | 0·19**(<0·001)** 0·13–0·27 | 0·21**(<0·001)** 0·14–0·32 | 0·74(0·227) 0·46–1·20 | 0·97(0·919) 0·56–1·69 |
| Attention, executive and memory impairments | 628(74) | 0·96(0·80) 0·72–1·30 | 1·23(0·30) 0·84–1·80 | 522(81) | 0·85(0·341) 0·61–1·19 | 0·73(0·098) 0·50–1·06 | 0·88(0·612) 0·55–1·43 | 0·56**(0·050)** 0·32–1·00 |
| Neurological and musculoskeletal impairments | 586(73) | 0·80(0·07) 0·63–1·02 | 0·75(0·06) 0·56–1·02 | 502(82) | 0·84(0·229) 0·63–1·12 | 0·77(0·122) 0·56–1·08 | 1·04(0·832) 0·70–1·56 | 1·07(0·763) 0·68–1·70 |
| Mental and emotional impairments | 389(75) | 1·16(0·37) 0·84–1·58 | 0·94(0·772) 0·64–1·40 | 309(85) | 1·38(0·100) 0·95–2·04 | 1·33(0·209) 0·86–2·08 | 1·19(0·513) 0·70–2·03 | 1·34(0·367) 0·71–2·51 |
| **Sensory impairments** |  |  |  |  |  |  |  |  |
| Blindness | 12(60) | 0·28**(0·006)** 0·12–0·69 | 0·19**(0·003)** 0·06–0·57 | 55(75) | 0·82**(<0·001)** 0·75–0·91 | 0·84**(0·006)** 0·75–0·96 | 2·91**(0·028)** 1·12–7·53 | 4·66**(0·014)** 1·37–15·83 |
| Visual impairment | 39(64) | 0·49**(0·02)** 0·27–0·91 | 0·40**(0·011)** 0·20–0·81 | 118(80) | 0·96**(0·002)** 0·93–0·99 | 0·97(0·055) 0·94–1·00 | 1·95**(0·037)** 1·04–3·65 | 2·49**(0·014)** 1·20–5·14 |
| Deaf-blindness | 20(67) | 0·42**(0·03)** 0·19–0·91 | 0·42(0·096) 0·15–1·17 | 25(78) | 0·92**(0·02)** 0·87–0·99 | 0·94(0·207) 0·87–1·04 | 2·22(0·056) 0·98–5·01 | 2·28(0·138) 0·77–6·78 |
| Deafness | 18(75) | 0·73(0·55) 0·26–2·03 | 1·81(0·43) 0·42–7·95 | 41(91) | 1·22(0·715) 0·47–4·22 | 0·91(0·865) 0·33–3·25 | 1·68(0·529) 0·34–8·38 | 0·49(0·493) 0·06–3·82 |
| Hearing impairment | 49(74) | 0·81(0·49) 0·45–1·47 | 1·14(0·72) 0·56–2·34 | 39(85) | 1·01(0·816) 0·95–1·07 | 1·01(0·680) 0·95–1·09 | 1·24(0·486) 0·67–2·29 | 0·89(0·764) 0·42–1·89 |
| Other impairments | 258(76) | 0·90(0·49) 0·67–1·22 | 0·83(0·30) 0·58–1·18 | 134(85) | 1·00(0·851) 0·99–1·01 | 1·00(0·827) 0·99–1·02 | 1·11(0·503) 0·82–1·50 | 1·22(0·265) 0·86–1·75 |
| Intercept * | 1006(93) | 5·98**(<0·001)** 5·26–6·79 | 7·12**(<0·001)** 5·00–10·17 | 1159(95) | 11·5**(<0·001)** 9·65–13·67 | 2·67**(<0·001)** 1·50–4·81 | 5·98**(<0·001)** 5·26–6·79 | 1·43(0·086) 0·95–2·16 |

* Reference group is participants without impairment, adjusted for gender (reference female) and age (reference <30 years of age). Further, for the interaction term models, reference is 2019 results as interaction terms with types of impairment. aOR = adjusted odds ratio; *P* = *P*-value; CI = 95% confidence interval.

## Table S8. Logistic regression models of avoiding booking health care appointments online, depending on types of impairment, year-stratified as well as comparison between the SMFOI19 and SMFOI21 surveys

|  | **Avoid booking health care appointments online 2019** | | | **Avoid booking health care appointments online 2021** | | | **SMFOI21 vs SMFOI19 Interaction term model** | |
| --- | --- | --- | --- | --- | --- | --- | --- | --- |
|  |  | n=1614 | n=1502 |  | n=1934 | n=1807 |  |  |
| **Independent variables** | n(%) | OR(*P*) CI | aOR(*P*) CI | n(%) | OR(*P*) CI | aOR(*P*) CI | OR(*P*) CI | aOR(*P*) CI |
| Language impairments | 151(54) | 1·60**(0·002)** 1·19–2·14 | 1·64**(0·003)** 1·19–2·27 | 100(35) | 1·22(0·195) 0·90–1·65 | 1·31(0·104) 0·94–1·82 | 0·77(0·225) 0·50–1·18 | 0·82(0·412) 0·51–1·32 |
| Intellectual impairments | 87(64) | 2·26**(<0·001)** 1·51–3·38 | 2·88**(<0·001)** 1·86–4·45 | 56(47) | 2·09**(0·001)** 1·37–3·16 | 2·74**(<0·001)** 1·74–4·33 | 0·92(0·793) 0·51–1·67 | 0·98(0·959) 0·51–1·90 |
| Attention, executive and memory impairments | 238(47) | 1·0(0·95) 0·76–1·35 | 1·10(0·54) 0·80–1·52 | 159(34) | 1·35**(0·04)** 1·01–1·80 | 1·75**(<0·001)** 1·27–2·40 | 1·34(0·173) 0·88–2·02 | 1·59**(0·047)** 1·01–2·52 |
| Neurological and musculoskeletal impairments | 211(44) | 1·0(0·99) 0·79–1·26 | 0·88(0·35) 0·68–1·14 | 132(30) | 0·88(0·353) 0·68–1·14 | 0·81(0·137) 0·61–1·07 | 0·89(0·508) 0·62–1·27 | 0·90(0·611) 0·62–1·33 |
| Mental and emotional impairments | 156(46) | 0·96(0·79) 0·70–1·30 | 1·25(0·21) 0·88–1·76 | 84(30) | 0·72(0·061) 0·51–1·01 | 0·81(0·258) 0·56–1·16 | 0·76(0·243) 0·47–1·21 | 0·66(0·111) 0·39–1·10 |
| **Sensory impairments** |  |  |  |  |  |  |  |  |
| Blindness | 4(67) | 3·45(0·16) 0·62–19·01 | 2·57(0·40) 0·29–22·83 | 35(73) | 1·41**(<0·001)** 1·27–1·58 | 1·38**(<0·001)** 1·22–1·57 | 0·41(0·395) 0·05–3·19 | 0·54(0·728) 0·02–17·78 |
| Visual impairment | 21(64) | 3·59**(0·001)** 1·64–7·85 | 5·40**(0·001)** 1·92–15·18 | 53(54) | 1·08**(<0·001)** 1·05–1·11 | 1·07**(<0·001)** 1·04–1·10 | 0·30**(0·004)** 0·13–0·68 | 0·19**(0·003)** 0·06–0·56 |
| Deaf-blindness | 9(47) | 1·35(0·52) 0·54–3·40 | 1·39(0·50) 0·54–3·60 | 9(41) | 1·05(0·146) 0·98–1·12 | 1·03(0·424) 0·95–1·12 | 0·78(0·615) 0·29–2·07 | 0·71(0·510) 0·26–1·96 |
| Deafness | 3(21) | 0·28(0·06) 0·07–1·07 | 0·37(0·18) 0·09–1·58 | 9(24) | 0·81(0·588) 0·35–1·68 | 0·97(0·952) 0·40–2·14 | 2·88(0·220) 0·53–15·60 | 2·77(0·295) 0·41–18·68 |
| Hearing impairment | 10(23) | 0·30**(0·001)** 0·15–0·62 | 0·25**(0·001)** 0·12–0·55 | 7(23) | 0·95(0·126) 0·89–1·01 | 0·93**(0·028)** 0·86–0·99 | 3·18**(0·003)** 1·49–6·77 | 3·68**(0·001)** 1·65–8·23 |
| Other impairments | 102(51) | 1·46**(0·02)** 1·07–1·99 | 1·26(0·17) 0·90–1·77 | 43(39) | 1·01(0·055) 1·00–1·02 | 1·01(0·131) 1·00–1·02 | 0·69**(0·023)** 0·51–0·95 | 0·79(0·182) 0·57–1·11 |
| Intercept * | 241(37) | 0·59**(<0·001)** 0·51–0·67 | 0·23**(<0·001)** 0·15–0·36 | 216(23) | 0·33**(<0·001)** 0·29–0·38 | 0·21**(<0·001)** 0·12–0·37 | 0·58**(<0·001)** 0·51–0·67 | 0·42**(<0·001)** 0·27–0·66 |

* Reference group is participants without impairment, adjusted for gender (reference female) and age (reference <30 years of age). Further, for the interaction term models, reference is 2019 results as interaction terms with types of impairment. aOR = adjusted odds ratio; *P* = *P*-value; CI = 95% confidence interval.

## Table S9. Logistic regression models of avoiding video health care appointments, depending on types of impairment (investigated only in the SMFOI21 survey)

|  | **Avoid video health care appointments** | | |
| --- | --- | --- | --- |
|  |  | n=453 | n=428 |
| **Independent variables** | n(%) | OR(*P*) CI | aOR(*P*) CI |
| Language impairments | 46(47) | 0·97(0·908) 0·59–1·59 | 1·07(0·809) 0·63–1·79 |
| Intellectual impairments | 21(58) | 1·78(0·139) 0·83–3·89 | 2·24(0·062) 0·98–5·34 |
| Attention, executive and memory impairments | 87(49) | 1·08(0·744) 0·67–1·74 | 1·02(0·942) 0·61–1·68 |
| Neurological and musculoskeletal impairments | 55(43) | 0·70(0·118) 0·44–1·09 | 0·68(0·118) 0·41–1·10 |
| Mental and emotional impairments | 57(52) | 1·30(0·317) 0·78–2·17 | 1·38(0·250) 0·80–2·40 |
| **Sensory impairments** |  |  |  |
| Blindness | 3(43) | 0·98(0·902) 0·75–1·27 | 0·87(0·469) 0·52–1·23 |
| Visual impairment | 6(38) | 0·97(0·443) 0·91–1·04 | 0·96(0·255) 0·89–1·03 |
| Deaf-blindness | 3(50) | 1·01(0·932) 0·88–1·15 | 0·98(0·792) 0·83–1·16 |
| Deafness | 4(36) | 0·68(0·552) 0·17–2·33 | 0·68(0·563) 0·17–2·40 |
| Hearing impairment | 1(25) | 0·96(0·564) 0·78–1·10 | 0·97(0·688) 0·79–1·12 |
| Other impairments | 22(59) | 1·02(0·086) 1·00–1·04 | 1·02**(0·042)** 1·00–1·04 |
| Intercept * | 77(48) | 0·78(0·074) 0·60–1·02 | 0·26**(0·014)** 0·08–0·75 |

* Reference group is participants without impairment, adjusted for gender (reference female) and age (reference <30 years of age). aOR = adjusted odds ratio; *P* = *P*-value; CI = 95% confidence interval.

## Table S10. Logistic regression models of difficulty in the use of the Swedish national web portal for health information and eHealth services (1177.se), depending on types of impairment, year-stratified as well as comparison between the SMFOI19 and SMFOI21 surveys

|  | **Difficulty in the use of the Swedish national web portal for health information and eHealth services (1177.se) in 2019** | | | **Difficulty in the use of the health information pages in the Swedish national web portal for health information and eHealth services (1177.se) in 2021** | | | **Difficulty in the use of the logged-in eHealth services in the Swedish national web portal for health information and eHealth services (1177.se) in 2021** | | | **Comparison between SMFOI21 and SMFOI19**  **Interaction term model** | |
| --- | --- | --- | --- | --- | --- | --- | --- | --- | --- | --- | --- |
|  |  | n=1618 | n=1534 |  | n=1773 | n=1681 |  | n=1828 | n=1736 |  |  |
| **Independent variables** | n(%) | OR(*P*) CI | aOR(*P*) CI | n(%) | OR(*P*) CI | aOR(*P*) CI | n(%) | OR(*P*) CI | aOR(*P*) CI | OR(*P*) CI | aOR(*P*) CI |
| Language impairments | 75(30) | 2·13**(<0·001)** 1·46–3·12 | 2·24**(<0·001)** 1·50–3·36 | 76(35) | 2·42**(<0·001)** 1·70–3·44 | 2·39**(<0·001)** 1·65–3·44 | 75(33) | 1·87**(<0·001)** 1·32–2·63 | 1·89**(<0·001)** 1·32–2·70 | 0·90(0·680) 0·54–1·49 | 0·89(0·659) 0·52–1·51 |
| Intellectual impairments | 26(26) | 0·88(0·65) 0·51–1·52 | 0·95(0·87) 0·53–1·71 | 34(40) | 1·63(0·061) 0·97–2·72 | 1·48(0·158) 0·85–2·53 | 33(38) | 1·49(0·118) 0·90–2·44 | 1·34(0·283) 0·78–2·25 | 1·80(0·110) 0·88–3·68 | 1·57(0·249) 0·73–3·39 |
| Attention, executive and memory impairments | 122(24) | 1·55**(0·02)** 1·06–2·27 | 1·55**(0·04)** 1·03–2·34 | 113(28) | 1·77**(0·001)** 1·25–2·49 | 1·82**(0·001)** 1·26–2·62 | 125(30) | 1·93**(<0·001)** 1·39–2·67 | 1·97**(<0·001)** 1·39–2·78 | 1·28(0·314) 0·79–2·08 | 1·48(0·138) 0·88–2·47 |
| Neurological and musculoskeletal impairments | 107(22) | 1·45**(0·02)** 1·05–1·99 | 1·34(0·09) 0·95–1·87 | 93(25) | 1·19(0·270) 0·87–1·62 | 1·17(0·343) 0·84–1·61 | 100(25) | 1·01(0·951) 0·75–1·35 | 0·96(0·796) 0·70–1·31 | 0·74(0·164) 0·49–1·13 | 0·69(0·097) 0·44–1·07 |
| Mental and emotional impairments | 78(24) | 1·14(0·49) 0·78–1·67 | 1·54**(0·04)** 1·019–2·316 | 58(23) | 0·72(0·102) 0·48–1·06 | 0·78(0·252) 0·51–1·19 | 62(25) | 0·73(0·105) 0·50–1·06 | 0·74(0·150) 0·49–1·11 | 0·62(0·068) 0·37–1·04 | 0·55**(0·029)** 0·32–0·94 |
| **Sensory impairments** |  |  |  |  |  |  |  |  |  |  |  |
| Blindness | 2(10) | 2·05(0·33) 0·49–8·62 | 1·88(0·42) 0·40–8·78 | 20(47) | 1·32**(<0·001)** 1·19–1·46 | 1·32**(<0·001)** 1·17–1·48 | 19(58) | 1·38**(<0·001)** 1·22–1·56 | 1·37**(<0·001)** 1·20–1·57 | 0·64(0·594) 0·13–3·26 | 0·64(0·603) 0·12–3·50 |
| Visual impairment | 6(21) | 1·25(0·67) 0·45–3·50 | 1·14(0·83) 0·35–3·72 | 33(43) | 1·10**(<0·001)** 1·06–1·13 | 1·09**(<0·001)** 1·06–1·13 | 34(37) | 1·07**(<0·001)** 1·04–1·10 | 1·07**(<0·001)** 1·04–1·11 | 0·86(0·786) 0·29–2·53 | 0·84(0·783) 0·25–2·85 |
| Deaf-blindness | 7(54) | 9·17**(<0·001)** 2·82–29·81 | 11·24**(<0·001)** 3·49–36·23 | 3(18) | 1·01(0·807) 0·90–1·11 | 1·04(0·516) 0·92–1·14 | 4(29) | 1·06(0·217) 0·96–1·16 | 1·08(0·126) 0·97–1·18 | 0·12**(<0·001)** 0·03–0·42 | 0·11**(<0·001)** 0·03–0·39 |
| Deafness | 4(25) | 2·24(0·21) 0·63–7·92 | 2·45(0·18) 0·66–9·13 | 10(27) | 2·08(0·059) 0·93–4·30 | 2·05(0·08) 0·87–4·41 | 6(17) | 0·95(0·919) 0·35–2·19 | 0·66(0·440) 0·19–1·72 | 0·64(0·579) 0·14–3·04 | 0·48(0·385) 0·09–2·51 |
| Hearing impairment | 13(32) | 2·68**(0·01)** 1·25–5·74 | 2·50**(0·02)** 1·17–5·35 | 6(19) | 0·97(0·384) 0·90–1·03 | 0·96(0·322) 0·89–1·03 | 5(17) | 0·96(0·245) 0·89–1·02 | 0·96(0·273) 0·89–1·03 | 0·37**(0·012)** 0·17–0·81 | 0·37**(0·014)** 0·16–0·82 |
| Other impairments | 51(26) | 1·64**(0·01)** 1·11–2·43 | 1·57**(0·03)** 1·03–2·39 | 25(25) | 1·01(0·419) 0·99–1·02 | 1·01(0·344) 0·99–1·02 | 27(27) | 1·01(0·387) 0·99–1·02 | 1·01(0·257) 0·99–1·02 | 0·61**(0·015)** 0·41–0·91 | 0·61**(0·020)** 0·40–0·92 |
| Intercept * | 46(7) | 0·10**(<0·001)** 0·08–0·12 | 0·05**(<0·001)** 0·02–0·08 | 100(11) | 0·14**(<0·001)** 0·12–0·17 | 0·14**(<0·001)** 0·07–0·28 | 126(13) | 0·17**(<0·001)** 0·15–0·20 | 0·20**(<0·001)** 0·10–0·38 | 0·10**(<0·001)** 0·08–0·12 | 0·09**(<0·001)** 0·05–0·15 |

* Reference group is participants without impairment, adjusted for gender (reference female) and age (reference <30 years of age). Further, for the interaction term models, reference is 2019 results as interaction terms with types of impairment. aOR = adjusted odds ratio; *P* = *P*-value; CI = 95% confidence interval.

## Table S11. Logistic regression models of difficulty in the use of digital identification, depending on types of impairment, year-stratified as well as comparison between the SMFOI19 and SMFOI21 surveys

|  | **Difficulty in the use of digital identification 2019** | | | **Difficulty in the use of digital identification 2021** | | | **Comparison between SMFOI21 and SMFOI19**  **Interaction term model** | |
| --- | --- | --- | --- | --- | --- | --- | --- | --- |
|  |  | n=2231 | n=2094 |  | n=2342 | n=2189 |  |  |
| **Independent variables** | n(%) | OR(*P*) CI | aOR(*P*) CI | n(%) | OR(*P*) CI | aOR(*P*) CI | OR(*P*) CI | aOR(*P*) CI |
| Language impairments | 76(22) | 1·71**(0·005)** 1·17–2·49 | 1·43(0·09) 0·95–2·16 | 47(15) | 3·72**(<0·001)** 2·26–6·10 | 3·18**(<0·001)** 1·87–5·37 | 2·18**(0·02)** 1·13–4·20 | 2·38**(0·017)** 1·17–4·83 |
| Intellectual impairments | 49(31) | 2·45**(<0·001)** 1·56–3·85 | 2·86**(<0·001)** 1·77–4·62 | 33(22) | 4·10**(<0·001)** 2·35–7·08 | 3·44**(<0·001)** 1·87–6·26 | 1·67(0·196) 0·77–3·64 | 1·52(0·330) 0·66–3·50 |
| Attention, executive and memory impairments | 121(19) | 1·90**(0·002)** 1·25–2·87 | 2·11**(0·001)** 1·33–3·34 | 56(10) | 2·13**(0·005)** 1·25–3·59 | 2·30**(0·004)** 1·30–4·02 | 1·12(0·758) 0·54–2·32 | 1·23(0·605) 0·57–2·65 |
| Neurological and musculoskeletal impairments | 92(15) | 1·16(0·410) 0·82–1·63 | 1·13(0·53) 0·78–1·64 | 40(8) | 0·90(0·682) 0·56–1·45 | 1·10(0·721) 0·65–1·83 | 0·78(0·435) 0·42–1·45 | 0·85(0·624) 0·44–1·63 |
| Mental and emotional impairments | 77(19) | 1·25(0·28) 0·84–1·86 | 1·47(0·08) 0·96–2·27 | 23(7) | 0·55**(0·043)** 0·30–0·97 | 0·51**(0·04)** 0·26–0·95 | 0·44**(0·034)** 0·20–0·94 | 0·38**(0·019)** 0·17–0·85 |
| **Sensory impairments** |  |  |  |  |  |  |  |  |
| Blindness | 2(20) | 3·51(0·13) 0·68–18·13 | 3·66(0·13) 0·67–19·94 | 16(26) | 1·57**(<0·001)** 1·40–1·75 | 1·65**(<0·001)** 1·45–1·87 | 0·45(0·389) 0·07–2·78 | 0·44(0·406) 0·07–3·02 |
| Visual impairment | 5(13) | 0·99(0·99) 0·33–3·00 | 1·03(0·96) 0·31–3·40 | 14(12) | 1·10**(<0·001)** 1·05–1·14 | 1·10**(<0·001)** 1·05–1·15 | 1·11(0·860) 0·35–3·49 | 1·01(0·989) 0·29–3·54 |
| Deaf-blindness | 6(29) | 5·01**(0·002)** 1·83–13·71 | 7·18**(<0·001)** 2·47–20·86 | 0(0) /n=27 | 0·36(0·976) 0·00–2·03 | 0·37(0·980) 0·00–4·28 | 0·08**(<0·001)** 0·03–0·23 | 0·06**(<0·001)** 0·02–0·18 |
| Deafness | 2(11) | 1·28(0·77) 0·24–6·71 | 1·40(0·71) 0·25–7·91 | 3(7) | 2·54(0·151) 0·57–7·83 | 1·77(0·463) 0·27–6·60 | 1·99(0·563) 0·19–20·31 | 1·44(0·782) 0·11–18·62 |
| Hearing impairment | 10(20) | 2·25**(0·03)** 1·10–4·62 | 2·21**(0·039)** 1·04–4·70 | 3(8) | 1·01(0·900) 0·91–1·09 | 0·99(0·912) 0·90–1·08 | 0·45**(0·034)** 0·21–0·94 | 0·43**(0·037)** 0·20–0·95 |
| Other impairments | 32(12) | 0·85(0·47) 0·54–1·33 | 0·81(0·39) 0·51–1·30 | 13(9) | 1·02(0·104) 1·00–1·03 | 1·02(0·052) 1·00–1·04 | 1·20(0·437) 0·76–1·88 | 1·19(0·472) 0·74–1·93 |
| Intercept * | 39(4) | 0·06**(<0·001)** 0·05–0·08 | 0·04**(<0·001)** 0·02–0·07 | 9(1) | 0·02**(<0·001)** 0·01–0·02 | 0·04**(<0·001)** 0·02–0·11 | 0·06**(<0·001)** 0·05–0·08 | 0·08**(<0·001)** 0·04–0·15 |

* Reference group is participants without impairment, adjusted for gender (reference female) and age (reference <30 years of age). Further, for the interaction term models, reference is 2019 results as interaction terms with types of impairment. aOR = adjusted odds ratio; *P* = *P*-value; CI = 95% confidence interval.
